# Percutaneous Pedicle Screw Placement Aided by a New Drill Guide Template Combined with Fluoroscopy: An Accuracy Study

**DOI:** 10.1111/os.12642

**Published:** 2020-03-04

**Authors:** Chao Wu, Jiayan Deng, Tao Li, Lun Tan, Dechao Yuan

**Affiliations:** ^1^ Represent Orthopedics Center of Zigong Fourth People's Hospital Zigong, Sichuan as province China; ^2^ Represent Digital Medical Center of Zigong Fourth People's Hospital Zigong, Sichuan as province China

**Keywords:** 3D printing technology, Minimally invasive spine surgery, Percutaneous pedicle screw fixation (PPS), Thoracolumbar fractures

## Abstract

**Objective:**

To evaluate the accuracy of percutaneous pedicle screw (PPS) placement aided by a new drill guide template.

**Methods:**

The patients were divided into guide template group and conventional perspective group. In the conventional perspective group, the screws were placed by hand under fluoroscopy. In the guide template group, the screw placement was aided by a new drill guide template, and the drill guide template is designed according to the patient's ideal pedicle screw, but not based on skin morphology. The accuracy was evaluated by comparing the following parameters between the two groups: pedicle breach level, inclination angle deviation between the left and right screws, sagittal angle deviation between the left and right screws, and position deviation of the left and right screw entry points. The consistency of the postoperative screw angle and the corresponding guide template inclination angle was compared in the guide template group. The operative time, blood loss, and radiation times were compared between the groups.

**Results:**

A total of 146 patients (876 screws) were enrolled in our study including 79 (474 screws) in the guide template group and 67 (402 screws) in the conventional perspective group. The pedicle breach level in the guide template group (22/474) was significantly lower than that in the conventional perspective group (47/402) (*P* < 0.05). The position and direction deviations of the left and right screws in the guide template group (2.06 ± 1.02 mm, 1.23 ± 1.25 mm, 1.83° ± 1.49°) were significantly less than those in the conventional perspective group (5.33 ± 2.99 mm, 4.32 ± 3.25 mm, 2.87° ± 1.56°). The operation time, blood loss, and radiation times were significantly lower in the guide template group (80.49 ± 9.14 min, 50.42 ± 8.9 mL, 11.02 ± 2.44) than those in the conventional perspective group (108.1 ± 21.18 min, 71.7 ± 17.09 mL, 23.53 ± 4.54). There were no significant differences between the postoperative screw angle and the corresponding guide template angle in the guide template group.

**Conclusion:**

PPS placement aided by a new drill guide template yielded higher screw accuracy and less operative time, blood loss, and radiation exposure than traditional screw placement.

## Introduction

Posterior pedicle screw placement is a common surgery in the treatment of thoracolumbar fractures^1^, ^2^. The traditional open procedure causes significant trauma and large tissue incisions. During the operation, the surrounding tissues are pulled for a long time, which affects the postoperative functional recovery of patients^3^, ^4^. With the development of minimally invasive spine surgery, percutaneous pedicle screw (PPS) fixation has been increasingly used by clinicians recently^5^, ^6^, ^7^, and determining how to confirm the pedicle screw entry point and orientation is a key issue associated with this technique^8^. The thoracolumbar spine has a complex anatomical structure and high mutation rate^9^. Conventional PPS surgery requires repeated adjust needles under perspective to ensure the safety of screw placement, which significantly increases the radiation exposure received by doctors and patients^10^. Although pedicle screw placement with limited touch by hand can reduce the need for fluoroscopy^11^, it can prolong the operative time, and it is difficult to ensure the consistency of the screw entry point and direction, which is not conducive to facilitating fracture reduction. Computer navigation can improve the accuracy of PPS placement^12^, but the high learning curve and high cost limit its application^13^, ^14^.

In recent years, a large number of studies have shown that 3D printed personal guide template can assist pedicle screw placement safely and accurately. However, the traditional navigation template is designed based on the surface morphology of the bone, so the skin and muscle need to be completely removed during the operation otherwise the accuracy of pedicle screw will be greatly reduced and even result in the possibility of breaking through the cortex. In addition, if the incision is not large enough it can decrease the accuracy of screw placement due to skin tension. Thus, a method to reduce radiation exposure and trauma while maintaining accurate PPS placement that is simple and easy to learn would be of great value.

In this study, we focused on improving the safety and accuracy of screw placement, reducing surgical trauma, reducing intraoperative radiation exposure, and minimizing surgical cost. As a consequence, we designed a complete extracellular porous structure navigation template, and applied a high‐precision 3D printer to make it; with the assistance of perspective, the pedicle screws can be implanted safety and accurately. The guide‐template‐assisted PPS placement designed in this study can also reduce trauma. Since we will accurately measure the pedicle angle based on the patient's computed tomography (CT) data before surgery, the navigation template suitable for the patient will be selected, which can avoid repeatedly adjusting the guide needle under fluoroscopy and effectively reduce the radiation exposure. Finally, the guide template designed in our study can be used repeatedly, and patients with similar pedicle angles can choose the same guide template, which greatly reduces the cost of 3D printed guide template.

The main purposes of this retrospective study include: (i) explore the feasibility of the guide template combined with fluoroscopy in PPS placement; (ii) investigate whether the guide template can reduce operation time, intraoperative bleeding, and radiation times in PPS implantation; and (iii) research the safety and accuracy of PPS placement assisted by the guide template.

## Materials and Methods

A retrospective analysis was performed on 146 patients enrolled in our research from February 2014 to December 2017. The patients were assigned to the conventional perspective group (n = 67) and guide template group (n = 79). There was no significant difference between the two groups in gender, age, body mass index (BMI), fracture segment, or AO (Arbeitsgemeinschaftfür Osteosynthesefragen) classification (Table 1). All patients underwent X‐ray and thin‐slice (<1 mm) CT imaging before and after surgery. All the patients understood the experimental design before the surgery and signed an informed consent form. This study was approved by the ethics committee of Zigong Fourth People's Hospital (No. 02, 2013).

**Table 1:** Patients characteristics (mean ± SD)

Clinical characteristics	Guide template group	Conventional perspective group	Statistics value	*P*
Cases	79	67		
Gender (male: female)	46:33	36:31	0.30	0.59
Age	48.91 ± 7.34	47.55 ± 8.32	1.04	0.30
BMI (body mass index)	24.80 ± 2.10	25.28 ± 2.66	−1.20	0.23
Fracture segment				
T_11_	9	7	0.16	0.98
T_12_	12	10		
L_1_	39	32		
L_2_	19	18		
AO classification				
A_1_	50	48	1.74	0.42
A_2_	9	8		
A_3_	20	11		

### *Participants*

The inclusion criteria were as follows: (i) thoracolumbar type A fractures; fracture located in T_11_ to L_2_; absence of neurological symptoms or signs; fracture occurrence within 1 week of study enrollment; (ii) in the guide template group, the screws were placed by fluoroscopy combined with a new guide template; (iii) in the conventional perspective group, the screws were placed by hand under fluoroscopy; and (iv) include outcomes: operation time, blood loss, incision length, and radiation times, grading criteria, screws symmetry and screw accuracy.

The exclusion criteria were as follows: (i) age greater than 60 years; (ii) fracture caused by pathological factors such as severe osteoporosis or tumor; (iii) patients with spinal deformity; (iv) patients with severe heart, liver and kidney diseases, diabetes, immune system diseases, or psychological disorders; and (v) patient follow‐up for less than 12 months.

### *Drill Guide Template*

**Figure 1:**
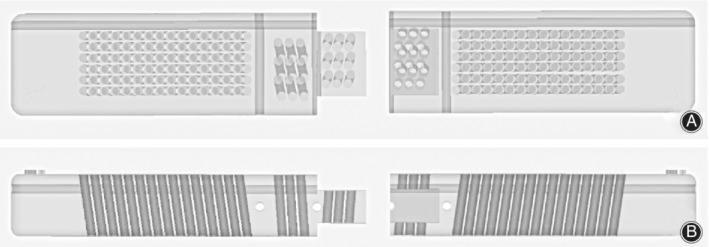
Sketch of the guide template. (A) The front view of the disassembled guide template. (B) The side view of the disassembled guide template.

A new sketch was generated in 3‐matic 13.0 (Materialize, Belgium). A rectangle with dimensions of 150 mm in length and 25 mm in width was drawn on the sketch, and the sketch was extended to a depth of 15 mm; then, a cuboid was generated (guide template). The guide template was divided into left and right parts and was connected through the buckle structure. The guide template was designed with a transverse hole running through the left and right sides to mark the upper edge of the vertebral body. The middle side of the guide template was designed with three longitudinal holes, which were used to pass through three K‐wires for connecting other guide templates, and were also used to locate the posterior spinous process, the left pedicle and the right pedicle. The left and right sides of the guide templates were designed with 6*15 holes, respectively, to guide the placement of the K‐wires, and all the holes with the same inclination angle. The left and right sides of the guide templates were designed with 3*3 holes, respectively, to locate the spinous process and fix the guide templates. The guide templates were classified into six types according to the inclination angle, which were 4°, 6°, 8°, 10°, 12°, and 14°. All the holes had a diameter of 2.1 mm, and a 2‐mm‐diameter K‐wire could pass through the holes easily. The guide templates were stored in STL format and imported into a 3DS 3600 printer (3D SYSTEMS company, America). The printing material was photosensitive resin (Fig. 1A, B).

### *Preoperative Measurement*

**Figure 2:**
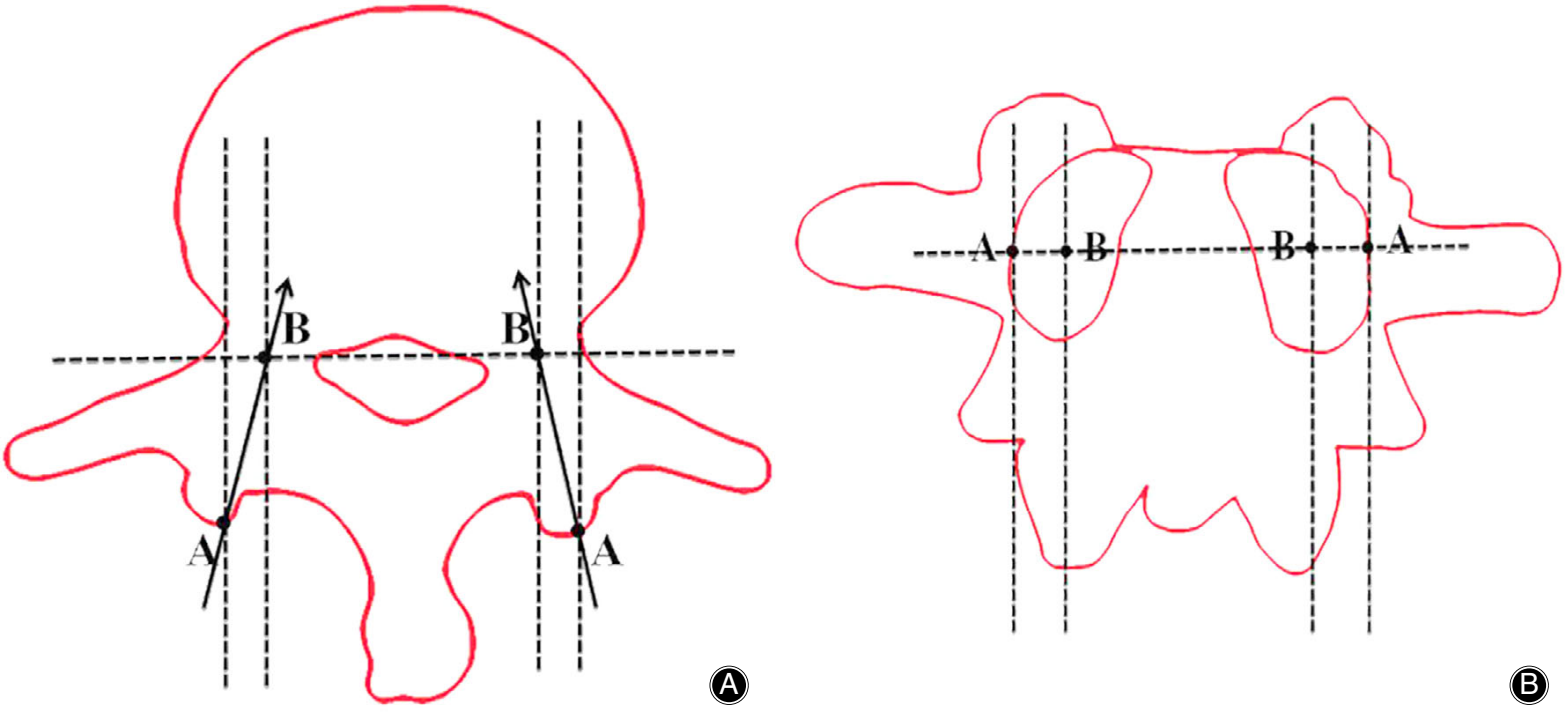
Measurement of the pedicle inclination angle. (A) The connection between point A and point B is an ideal pedicle screw path in the axial view. The angle between the connection line and the sagittal plane was regarded as the inclination angle. (B) In the anteroposterior view, point A is at the pedicle margin and point B is at the pedicle center.

Two points (point A and point B) were selected on the axial vertebral. Point A was the junction of the extension of the lateral margin of the pedicle and articular process, and point B was the center of the narrowest pedicle. The connection line between point A and point B was recognized as the optimal screw channel, and the angle between the connection line and the sagittal plane was the inclination angle of the pedicle screw (Fig. 2A, B).

### *Surgery*

All the surgeries were performed by one experienced surgeon. Under general anesthesia, the patient was placed in the prone position, avoiding pressure on the abdomen.

#### *Guide Template Group*

##### Approach and Exposure

The vertebral body surface projections of the injured vertebrae and the adjacent vertebrae were located and marked with the aid of a C‐arm machine. The pedicle inclination angle of the target vertebrae was measured according to the method described in Fig. 2 before the operation. The most suitable drill guide template among the above six different templates according to inclination angle of the vertebra was selected and then sterilized.

##### Reconstruction

**Figure 3:**
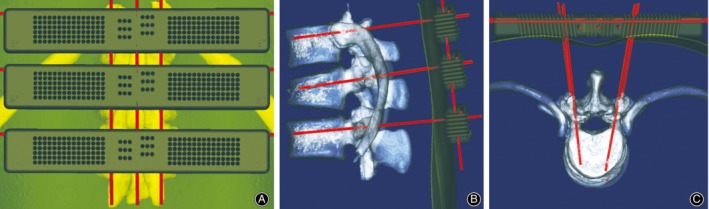
Surgical procedure simulation in a male patient with an L_1_ fracture. (A) Assembly of the guide templates outside the skin. (B, C) Simulation of K‐wire placement under the guide template.

**Figure 4:**
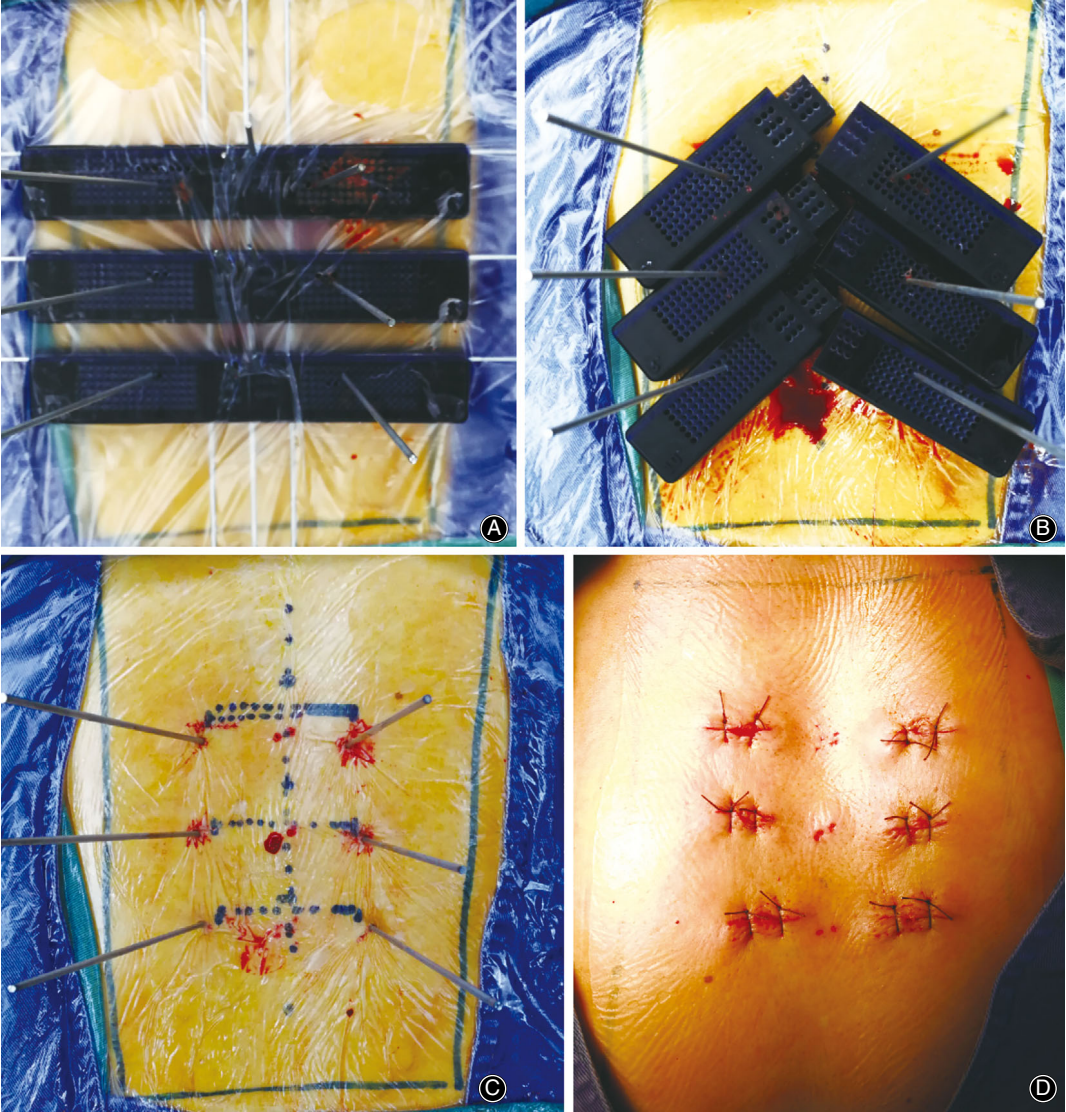
Surgical procedure in the above male patient. (A) The guide template was fixed on the skin surface, and K‐wires were placed through the guide template. (B) The disassembled guide template. (C) The guide template was removed, and the K‐wires are shown. (D) Stitched wound after pedicle screw placement.

**Figure 5:**
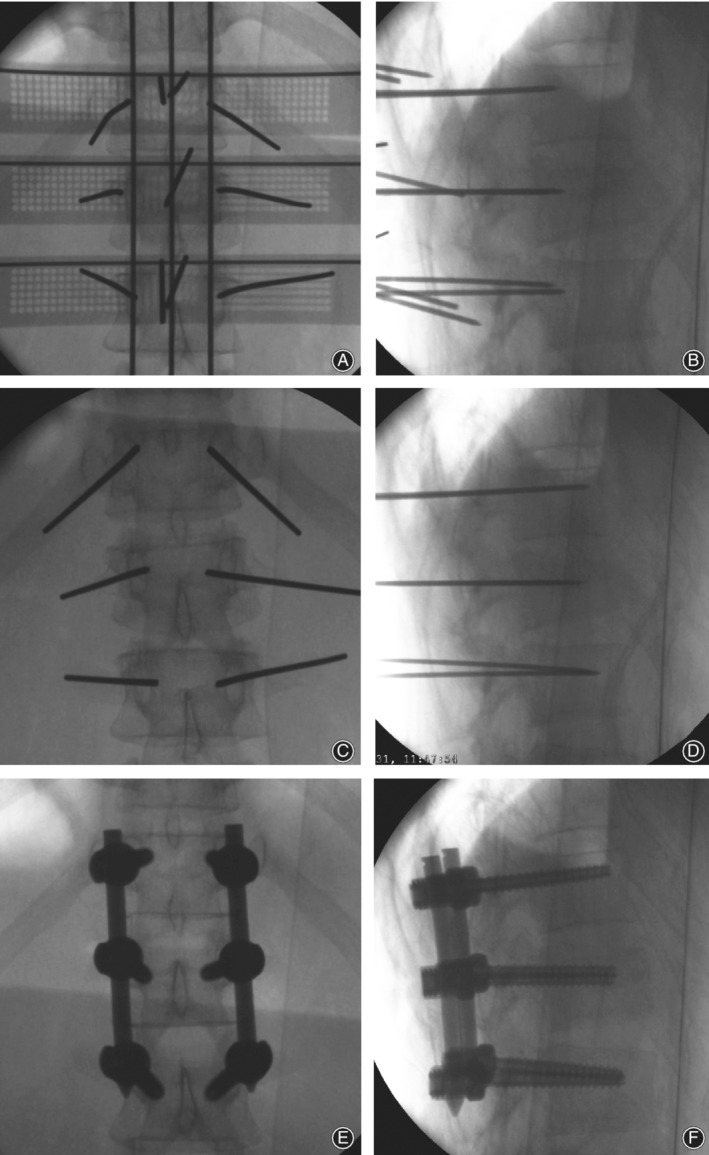
Intraoperative C‐arm fluoroscopic images of the above patient. (A, B) After the K‐wires were inserted into the pedicle, the tip of the K‐wire did not exceed the medial margin of the pedicle in the C‐arm fluoroscopy. (C, D) The K‐wires were inserted into the front 1/3 of the vertebral body in lateral fluoroscopy and lateral fluoroscopy. (E‐F) The screw was placed to reduce the fracture and fix the screw.

During the operation, the left and right parts were first assembled. Three K‐wires with diameter of 2 mm were selected and horizontally penetrated into the transverse fluoroscopy positioning holes of the guide templates. Then, three K‐wires were penetrated into the vertical fluoroscopy positioning holes to connect the guide templates as a whole (Fig. 3A). A standard antero‐posterior radiograph confirmed that the K‐wires were in suitable positions (the horizontal K‐wires were located at the upper edge of each vertebral body, and the longitudinal K‐wires were located along the line of the spinous process and the line of the projected center of the pedicle on both sides). The guide templates were fixed with 2‐mm‐diameter K‐wires through the spinous process positioning hole. A suitable guide hole on the guide template was selected based on an antero‐posterior photograph, and six K‐wires were punched by a hammer through the guide hole (Fig. 4A). After reaching the bone, standard antero‐posterior and lateral radiography was performed (Fig. 5A), and the position of the K‐wires was adjusted according to the radiographs before the K‐wire penetrated into the pedicle. The K‐wire tip was located at the outer edge of the pedicle (antero‐posterior fluoroscopy) (Fig. 5B). When the K‐wire tip reached the posterior border of the vertebra (lateral radiograph), the tip of the K‐wire did not exceed the medial margin of the vertebral pedicle (antero‐posterior fluoroscopy). During the penetration, the K‐wire was parallel to the upper endplate of the vertebral body on the lateral radiograph, and the tip of the K‐wire finally reached the front 1/3 of the vertebra (Fig. 3B, C, Fig. 5C, D). The left and right guide plates are separated and removed (Fig. 4B). The following steps were the same as those used in the conventional method (Fig. 4C, D, Fig. 5E, F).

#### *Conventional Perspective Group*

This group underwent PPS placement aided by conventional fluoroscopy alone. This procedure was performed as described by Heintel TM *et al*.^15^.

All the patients left their bed and were supported by a hard brace at 5 days postoperatively. The hard brace was worn for 12 weeks. The pedicle screws were removed 6 to 12 months after surgery with CT confirmation of solid fusion.

### *Data Collection and Outcome Evaluation*

The observation data for two groups were recorded, the clinical parameter for two groups were measured and analyzed.

#### *Operative Time*

In our study, the operation time was defined as the period between the incision and the safe placement of the screws. Less operation time can effectively reduce the operation risk.

#### *Blood Loss*

The amount of blood loss during the operation can reflect the quality of the operation.

A small amount of blood loss helps speed the patient's recovery.

#### *Radiation Times*

Radiation time represents to the number of c‐arm fluoroscopy during the whole operation. Fewer radiation times can reduce the radiation exposure to operators and patients.

#### *Grading Criteria*

Grading is an important parameter to evaluate screw safety. Postoperative CT images were obtained to assess the accuracy of screw placement for each patient. Screw breach of the pedicle cortex in the axial planes was evaluated with a grading scale in 2‐mm steps (grade I: no breach; grade II: breach less than 2 mm; grade III: breach 2 to 4 mm; grade IV: breach greater than 4 mm), as described by Gertzbein and Robbins^16^.

#### *Screw Symmetry*

Screw symmetry refers to the consistency of left and right screw placement. The bilateral screw consistency were measured between two groups by comparing the following parameters: the absolute inclination angle deviation between bilateral screws and the absolute sagittal angle deviation between bilateral screws. The entry point deviation between bilateral screws. Screw symmetry reflects the difference between screw placement and ideal pedicle screw channel.

#### *Screw Accuracy*

The screw accuracy refers to the deviation of screw entry point and screw orientation between postoperative and preoperative screw placement. The postoperative inclination angle was compared with the corresponding guide template in the guide template group. The smaller the deviation, the higher the accuracy of drill guide template‐assisted pedicle screw placement.

### *Statistics*

All statistical analyses were performed in SPSS 19.0 (SPSS Inc.; Chicago, IL, USA). Means and standard deviations of every parameter were calculated to two decimal places. The paired‐sample student's *t*‐test was conducted for intragroup comparison, including inclination angle, and an independent two‐sample student's *t*‐test was conducted for intergroup comparison, including age, BMI, operation time, blood loss, incision length, radiation times, and bilateral screw angle consistency. The chi‐square test was performed for enumeration data, such as gender, fracture segment, and AO classification. The Kruskal‐Wallis test was performed for rank data, such as grading. The level of statistical significance was set at 0.05 for all statistical analyses.

## Results

### *General Results*

All patients underwent surgery successfully, and a total of 876 pedicle screws were placed. One patient developed a superficial infection in each group, which healed after wound dressing and antibiotic treatment. No significant vascular or nerve injury was found, and no screw loosening or crack resulting from internal fixation was found at the last follow‐up. All patients were followed up for 12 to 24 months. There was no significant difference between the two groups in gender, age, BMI, fracture segment, and AO classification.

### *Intra‐operative Results*

The operation time in the guide template group of 80.49 ± 9.14 min was significantly lower than that of 108.10 ± 21.18 min in the conventional perspective group; the blood loss in the guide template group of 50.42 ± 8.90 mL was significantly lower than that of 71.70 ± 17.09 mL in the conventional perspective group; the radiation time in the guide template group of 11.02 ± 2.44 was significantly lower than that of 23.53 ± 4.54 in the conventional perspective group (Table 2).

**Table 2:** Comparisons of the perioperative parameters between the two groups (mean ± SD, n = 146)

Item	Guide template group	Conventional perspective group	*t*	*p*
Operation time (min)	80.49 ± 9.14	108.10 ± 21.18	−9.92	<0.05
Blood loss (mL)	50.42 ± 8.90	71.70 ± 17.09	−9.19	<0.05
Incision length (cm)	12.29 ± 3.39	12.42 ± 5.51	−0.17	0.87
Radiation times	11.02 ± 2.44	23.53 ± 4.54	−20.04	<0.05

### *Safety of Screws*

In the guide template group, there were 452 screws classified as grade 0, 20 screws classified as grade 1, two screws classified as grade 2, and no screws classified as grade 3. In the conventional perspective group, there were 355 screws classified as grade 0, 26 screws classified as grade 1, 12 screws classified as grade 2, and 9 screws classified as grade 3. The screw breaches of the pedicle cortex in the axial planes were significantly different between the two groups (*P* < 0.05) (Table 3).

**Table 3:** Classification of pedicle screws

Grade	Guide template group	Conventional perspective group	*z*	*P*
n	474	402		
Grade I	452	355	−3.97	<0.05
Grade II	20	26		
Grade III	2	12		
Grade IV	0	9		

### *Symmetry of Screws*

The average absolute deviation in the inclination angle between the left and right screws was 2.06° in the guide template group and 5.33° in the conventional perspective group, corresponding to a significant difference between the two groups (*P* < 0.05).

The average absolute deviation in the sagittal angle between the left and right screws was 1.23° in the guide template group and 4.32° in the conventional perspective group, corresponding to a significant difference between the two groups (*P* < 0.05).

The average position deviation between the left and right screw entry points was 1.83 mm in the guide template group and 2.87 mm in the conventional perspective group, corresponding to a significant difference between the two groups (*P* < 0.05) (Table 4).

**Table 4:** Bilateral screw angle symmetry comparison between two groups

Variables	Guide template group	Conventional perspective group	*t*	*P*
n	237	201		
Inclination angle absolute deviation between bilateral screws (°)	2.06 ± 1.02	5.33 ± 2.99	−16.66	<0.05
Sagittal angle absolute deviation between bilateral screws (°)	1.23 ± 1.25	4.32 ± 3.25	−15.02	<0.05
Position deviation of screw entry point (mm)	1.83 ± 1.49	2.87 ± 1.56	−7.05	<0.05

### *Accuracy of Screws*

**Figure 6:**
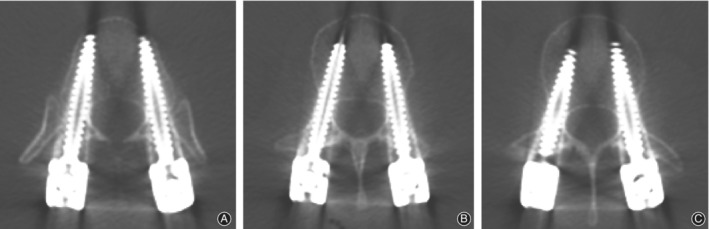
CT images of the above patient. (A) Postoperative CT axial images of T_12_. (B) Postoperative CT axial images of L_1_. (C) Postoperative CT axial images of L_2_.

In the guide template group, a 4° guide template was used for 42 screws, a 6° guide template was used for 18 screws, an 8° guide template was used for 160 screws, a 10° guide template was used for 130 screws, a 12° guide template was used for 96 screws, and a 14° guide template was used for 28 screws. There were no significant differences in the inclination angle between all screws and the corresponding guide templates *(P* > 0.05) (Table 5, Fig. 6).

**Table 5:** The postoperative inclination angle was compared with the corresponding angle of guide template in guide template group

Guide classification	Screws	Post‐operative inclination angle (°)	*t*	*P*
4°	42	4.08 ± 0.33	1.52	0.14
6°	18	6.43 ± 0.92	2.00	0.06
8°	160	8.26 ± 1.72	1.90	0.06
10°	130	10.35 ± 2.28	1.77	0.08
12°	96	12.30 ± 2.55	1.15	0.25
14°	28	14.46 ± 2.02	1.22	0.24

## Discussion

### *General Treatment for PPS Placement*

Posterior pedicle screw fixation is widely used in the treatment of thoracolumbar fractures. Although the open posterior approach surgery can effectively fix the fractured vertebra, promote fracture healing, and reduce complications, it results in large incisions, significant blood loss, destruction of peripheral soft tissue, and complications such as back pain^17^, ^18^, ^19^. The PPS system has obvious advantages in reducing soft tissue injury and postoperative pain^20^, ^21^.

### *Advantages of a New Drill Guide Template Assisting PPS Placement*

In this study, we designed and applied the new guide plate outside the skin in clinical practice and achieved good results. The main advantages of the new guide plate were as follows. (i) Improvement of screw placement accuracy: the preoperative inclination angle was measured, and the corresponding guide template was selected. The guide plate was positioned through fluoroscopy, and it was placed outside the skin through K‐wires to avoid errors caused by skin movement. Regarding the standard fluoroscopy procedure, the guide‐template‐fluoroscopy K‐wires were located in the upper endplate and between the connecting line of the spinous process; the other fluoroscopy K‐wires were located in the connecting line of the projected center of the bilateral pedicle (the spinous process was located in the middle of the bilateral pedicle) to ensure that the K‐wires' inclination were consistent with the preoperative design. Multiple optional puncture holes in the guide template can aid in accurately positioning the screw entry point and the K‐wires were easily adjusted objectively. In the guide template group, a total of 452 screws were positioned entirely within the pedicle cortex, which was a significantly better result than that achieved in the conventional perspective group. (ii) A guide template with good stability: Different guides are connected by three K‐wires in the same plane, which can increase the stability of the relative position between different guides and ensure that K‐wires can be adjusted in the sagittal plane. (iii) A bilateral pedicle screw with better consistency: The left and right orientation holes of the guide template have the same inclination angle. In the guide template group, the average inclination angle deviation of the left and right sides was 2.06 mm. (iv) Shorter operation time and fluoroscopy exposure: In this study, six positioning needles were accurately inserted at one time in the guide template group and the operation time was shortened. In addition, the guide template composed of a photosensitive resin was developed under fluoroscopy, and could be adjusted under fluoroscopy, thus reducing the fluoroscopy time.

### *Surgical Tips*

We provide the following tips to improve the accuracy of PPS placement based on the experience gained in this study: (i) in order to reduce intraoperative adjustment of the guide plate, preoperative fluoroscopy was performed to mark the surface location of the upper endplate, spinous process and pedicle; (ii) after determining the position of the guide template, in addition to the fixation of the spinous process with the positioning needle, the fixation can also be strengthened with the surgical film (3M Steri‐Drape); (iii) the guide template ensures that the connecting line of the entry point of the positioning wires is rectilinear to facilitate the placement of the connecting wires; and (iv) because the guide plates are connected with K‐wires, the sagittal orientation of the pedicle‐guide K‐wires can be adjusted again under fluoroscopy.

### *Limitation*

First, the guide templates were classified into six types according to the inclination angle, which were 4°, 6°, 8°, 10°, 12°, and 14° in this study; in order to make the guide template suitable for more patients, we will expand the inclination range of guide template. In order to improve the accuracy of guide‐plate‐assisted screw placement, we would shorten the inclination angle interval step. In future studies, we will design more types of guide templates. Second, this study focused on the safety and accuracy of guide template‐assisted PPS. The functional outcomes were not analyzed in this study and we will integrate the functional outcomes into research in the future.

### *Conclusion*

The use of guide‐template‐assisted PPS placement resulted in less bleeding, less radiation, and greater screw positioning accuracy. The structure of the guide template is simple, and the guide template is easy to operate. Further research on this technique deserves consideration.
